# Fate of Articles That Warranted Retraction Due to Ethical Concerns: A Descriptive Cross-Sectional Study

**DOI:** 10.1371/journal.pone.0085846

**Published:** 2014-01-22

**Authors:** Nadia Elia, Elizabeth Wager, Martin R. Tramèr

**Affiliations:** 1 Division of Anaesthesiology, Geneva University Hospitals, and Medical Faculty, University of Geneva, Geneva, Switzerland; 2 Institute of Social and Preventive Medicine, Medical Faculty, Geneva, Switzerland; 3 Sideview, Princes Risborough, United Kingdom; State University of New York, Oswego, United States of America

## Abstract

**Objective:**

To study journals' responses to a request from the State Medical Association of Rheinland-Pfalz, Germany, to retract 88 articles due to ethical concerns, and to check whether the resulting retractions followed published guidelines.

**Design:**

Descriptive cross-sectional study.

**Population:**

88 articles (18 journals) by the anaesthesiologist Dr. Boldt, that warranted retraction.

**Method:**

According to the recommendations of the Committee on Publication Ethics, we regarded a retraction as adequate when a retraction notice was published, linked to the retracted article, identified the title and authors of the retracted article in its heading, explained the reason and who took responsibility for the retraction, and when the retracted article was freely accessible and marked using a transparent watermark that preserved original content. Two authors extracted data independently (January 2013) and contacted editors-in-chief and publishers for clarification in cases of inadequate retraction.

**Results:**

Five articles (6%) fulfilled all criteria for adequate retraction. Nine (10%) were not retracted (no retraction notice published, full text article not marked). 79 (90%) retraction notices were published, 76 (86%) were freely accessible, but only 15 (17%) were complete. 73 (83%) full text articles were marked as retracted, of which 14 (16%) had an opaque watermark hiding parts of the original content, and 11 (13%) had all original content deleted. 59 (67%) retracted articles were freely accessible. One editor-in-chief stated personal problems as a reason for incomplete retractions, eight blamed their publishers. Two publishers cited legal threats from Dr. Boldt's co-authors which prevented them from retracting articles.

**Conclusion:**

Guidelines for retracting articles are incompletely followed. The role of publishers in the retraction process needs to be clarified and standards are needed on marking retracted articles. It remains unclear who should check that retractions are done properly. Legal safeguards are required to allow retraction of articles against the wishes of authors.

## Introduction

On the 25^th^ of February 2011, the State Medical Association of Rheinland-Pfalz, Germany, informed all affected medical journals of the results of its evaluation regarding the status of Institutional Review Board approval for research conducted by the anaesthetist Dr. Joachim Boldt. The evaluation revealed that 88 original articles authored by Dr. Boldt, and published in 18 peer-reviewed journals, lacked formal ethical approval. As a consequence, the editors-in-chief of these 18 journals signed a common statement declaring their intention to retract these articles from their journals, and to publish formal retraction notices [1].

Authoritative bodies such as the Committee On Publication Ethics (COPE) [2], and the National Library of Medicine (NLM) [3] have produced guidelines concerning the retraction of fraudulent research papers. According to COPE, retraction notices should be published and linked to the retracted article, be clearly identified as a retraction (not as a correction or a comment), identify the retracted article by including the title and authors in the retraction heading, explain who is retracting and the reasons for retraction, and finally, be freely accessible to all reader, thus not be hidden behind access barriers [4]. NLM adds a statement stipulating that “*The retraction should appear on a numbered page in a prominent section in an issue of the print journal that published the retracted article as well as in the online version, be listed in the Table of Contents page.”* NLM does not remove the original reference of a retracted article, but updates the citation to indicate it has been retracted and adds a link to the retraction statement. The original article should be retained unchanged, except for a watermark on the PDF indicating on each page that it is “retracted”. Sox and Rennie have further recommended that, as with retraction notices, journals should provide free access to the full text of retracted fraudulent articles [5].

In earlier cases, some occurring before these recommendations were published, journals were found not to have retracted articles appropriately [5], [6]. Although the importance of appropriately retracting fraudulent articles has often been underlined in Commentaries and Editorials [7]–[9], studies attempting to quantify the problem are still lacking. We therefore set out to study the fate of the 88 articles by Dr. Joachim Boldt that were meant to be retracted in early summer 2011 because of lack of ethical approval.

## Methods

This study is reported according to the STROBE statement for reporting cross-sectional studies.

### Ethics statement

Ethical approval was not required for this study.

### Study design and setting

This was a descriptive cross-sectional study. All searches and data extraction were done in January 2013.

### Study selection

We selected all 88 articles listed in the document *“Editors-in-Chief Statement Regarding Published Clinical Trials Conducted without IRB Approval by Joachim Boldt”* (published in February 2011) for which the State Medical Association of Rheinland-Pfalz, Germany, was unable to verify approval by a competent ethics committee and had therefore recommended that they should be retracted [1].

### Variables, data extraction and data sources

For each article, we checked whether all criteria for adequate retraction, as stipulated by COPE, were fulfilled [2]. In addition (following other recommendations) we checked whether the full text article was freely accessible [5], and whether the original content was preserved [10].

For each title, two authors (NE, MRT) independently recorded whether or not a retraction notice had been published in the respective journal (*yes, no*), whether the retraction notice was linked to the retracted article (*yes, no*), was listed in the journal's table of contents (*completely*: complete reference of retracted article is listed in table of contents; *incompletely*: retraction is listed in table of contents but reference is lacking; *none*: retraction notice is not listed), and whether the heading of the retraction notice included the title (*yes, no*) and the authors (*yes, no*) of the retracted article.

For each retraction notice that could be retrieved, we checked whether or not the notice provided explanations concerning the reason for retraction (*yes, no*), and described who was responsible for the retraction (*editor-in-chief, editorial board, publisher*, etc).

We checked on the article PDFs whether, and how, they were marked, for instance, using a watermark indicating “retracted” across all pages. We classified the watermarks as *transparent* (i.e. underlying text, figures or tables were readable) or *opaque* (i.e. underlying text, figures and tables were obscured).

In order to assess the accessibility of retraction notices and full text retracted articles, all searches were performed from a private computer without subscription to any journal. For the searches of the full text PDF, we copied the titles of retracted articles into Google®. If a link to Pubmed was provided, that link was tried first. If a link to the full text was provided in Pubmed, that link was used. If the Pubmed link led to the full text (online or PDF), the article was classified as *freely accessible through Pubmed*. If the Pubmed link led to a login page requiring registration and/or a fee for access to the article, the article was classified as *not freely accessible through Pubmed*. For all articles that were not freely accessible through Pubmed, alternative links provided through Google were searched (for instance, ScienceDirect [http://www.sciencedirect.com/], ResearchGate [http://www.researchgate.net/] or Google Scholar [http://scholar.google.ch/]). When this secondary search was successful, the article was classified as *freely accessible through alternative web sources*. If it was not, it was classified as *not freely accessible through alternative web sources*. The same procedure was repeated to assess accessibility of retraction notices.

We considered a retraction as *adequate* if the following criteria were fulfilled: a retraction notice was published and linked to the retracted article, was identified as a “retraction” in the table of contents of the journal, included title and authors of the retracted article in its heading, explained who took responsibility for the retraction, gave reasons for retraction, and was freely accessible, and if the PDF of the retracted article was clearly labelled as “retracted” using a transparent watermark preserving original content and was freely accessible.

Finally, we contacted the editors-in-chief of those journals that had failed to retract articles correctly, and asked them for an explanation. If feasible, we also contacted the journal publishers and asked for explanations.

### Bias

Data extraction was performed by two authors (NE and MT) independently in order to minimise the risk of extraction errors. Clear procedures were defined before performing the searches in order to guarantee reproducibility of the findings.

### Study size and statistical analyses

The study sample was defined as all trials that were authored or co-authored by Dr. Joachim Boldt and that were found by an official inquiry to warrant retraction because of lack of formal ethical approval. This is a descriptive study; there was no intention to search for associations between variables or to draw statistical inferences; therefore, no sample size calculation was performed. Results are reported as frequencies and percentages.

## Results

### Journals and publishers

The 88 articles were published in *Anesthesia and Analgesia* (22 articles), *British Journal of Anaesthesia* (11), *Journal of Cardiothoracic and Vascular Anesthesia* (9), *European Journal of Anaesthesiology* (8), *Anaesthesia* (6), *Anästhesiologie Intensivmedizin Notfallmedizin Schmerztherapie* (6), *Canadian Journal of Anesthesia* (5), *Intensive Care Medicine* (5), *Acta Anaesthesiologica Scandinavica* (3), *Der Anästhesist* (2), *Annals of Thoracic Surgery* (2), *Critical Care Medicine* (2), *Thoracic and Cardiovascular Surgery* (2), *Medical Science Monitor* (2), *Minerva Anestesiologica* (2), *Anesthesiology* (1), *Journal of Cranio-Maxillo-Facial Surgery* (1), and *Vox Sanguinis* (1) (Table 1).

**Table 1:** Summary of 88 articles that warranted retraction.

Journal and publisher	Retractions	Retraction notice											Full text article (PDF)			
							Heading includes		Notice describes		Free access		Watermark		Free access	
		Published	Month	Linked to article	Identified as such	In table of content	Article title	Authors	Who retracts	Reasons	Pubmed	Other	Present	Quality	Pubmed	Other
***Acta Anaesthesiologica Scandinavica***																
Wiley-Blackwell	3	3	Aug	YES	YES	YES^a^	NO	NO	YES	YES	YES		NO		NO	YES (1)
***Anaesthesia***																
Wiley-Blackwell	6	6	July	YES	YES	YES^a^	NO	NO	YES	YES	YES		YES	Transparent	YES (5)	YES (1)
***Anästhesiologie Intensivmedizin Notfallmedizin Schmerztherapie***																
Thieme	6	YES (2)	July	YES	YES	NO	YES	NO	YES	YES	YES		YES (2)	Opaque	NO	NO
		NO (4)											NO (4)		NO	NO
***Anesthesia & Analgesia***																
Lippincott Williams & Wilkins	22	22	May	YES	NO	YES^a^	YES	NO	YES	NO^c^	YES		YES	Transparent	YES	
***Anesthesiology***																
Lippincott Williams & Wilkins	1	1	May	YES	YES	YES^b^	NO	NO	NO	YES	YES		YES	Transparent	YES	
***Annals of Thoracic Surgery***																
Elsevier	2	2	July	YES	YES	YES^a^	NO	NO	YES	YES	YES		YES	Opaque	YES (1)	NO
***British Journal of Anaesthesia***																
Oxford University Press	11	11^col^	July	YES	YES	YES^b^	NO	YES	YES	YES	YES		YES	Content deleted^e^	YES (empty)	
***Canadian Journal of Anesthesia/Journal canadien d'anesthésie***																
Springer	5	5	Sept	YES	YES	YES^a^	YES	YES	YES	YES	YES		YES	Transparent^f^	YES (4)	
															NO (1)	YES (1)
***Critical Care Medicine***																
Lippincott Williams & Wilkins	2	2^col^	Oct	YES	YES	YES^b^	NO	NO	NO	YES	NO	NO	YES (1)	Transparent	NO	NO
													NO (1)		NO	NO
***Der Anästhesist***																
Springer	2	YES (1)	May	YES	NO^1^	NO	YES	NO	YES	YES	NO	NO	YES (1)	Opaque	NO	NO
		NO (1)											NO (1)		NO	NO
***European Journal of Anaesthesiology***																
Lippincott Williams & Wilkins	7	7	June	YES	YES	YES^a^	YES	NO	YES	NO^c^	YES		YES	Transparent	NO	NO
Cambridge University Press	1	1	June	YES	YES	YES^a^	YES	NO	YES	NO^c^	YES		NO ^d^		NO	NO
***Intensive Care Medicine***																
Springer	5	5	July	YES	YES	YES^a^	YES	YES	NO	YES	YES		YES	Transparent^f^	NO	NO
***Journal of Cardiothoracic and Vascular Anesthesia***																
W.B. Saunders Company; Elsevier	9	9	Aug	YES	YES	YES^b^	YES	YES	YES	YES	NO	YES	YES	Opaque	NO	YES
***Journal of Cranio-Maxillofacial Surgery***																
Thieme; Elsevier	1	None											NO		NO	NO
***Medical Science Monitor***																
Medical Science International	2	None											NO		NO	YES
***Minerva Anestesiologica***																
Edizioni Minerva Medica	1	1	May	YES	NO^2^	YES^b^	NO	YES	YES	YES	YES		NO		NO	YES
***The Thoracic and Cardiovascular Surgeon***																
Thieme	1	None											NO		NO	NO
***Vox Sanguinis***																
Wiley-Blackwell	1	1	July	YES	YES	YES^a^	YES	YES	YES	YES	YES		YES	Transparent	NO	NO

Numbers in parentheses are numbers of articles or of retraction notices. ^Col^collective notice of retraction published. ^1^erratum; ^2^statement. ^a^complete reference of retracted article is listed in table of content; ^b^retraction is listed in table of content but reference is lacking; ^c^reason not described in notice but notice refers to an editorial giving the reason; ^d^PDF of one article that was published by a former publisher not marked; ^e^PDF accessible but original text, figures, tables deleted; ^f^Watermark present but almost invisible. Column “Month” refers to year 2011. Since January 2013, one publisher (Wiley-Blackwell) has modified its retraction notices after learning of our findings.

The 18 journals were published by nine publishers (Table 1): Lippincott Williams & Wilkins, Wiley-Blackwell, Springer, and Elsevier (three journals each), Thieme (2), Oxford University Press, Edizioni Minerva Medica and Medical Science International (1 each). One journal (*European Journal of Anaesthesiology*) had changed publishers in 2009 (from Cambridge University Press to Lippincott Williams & Wilkins).

### Outcome data and main results

**Figure 1:**
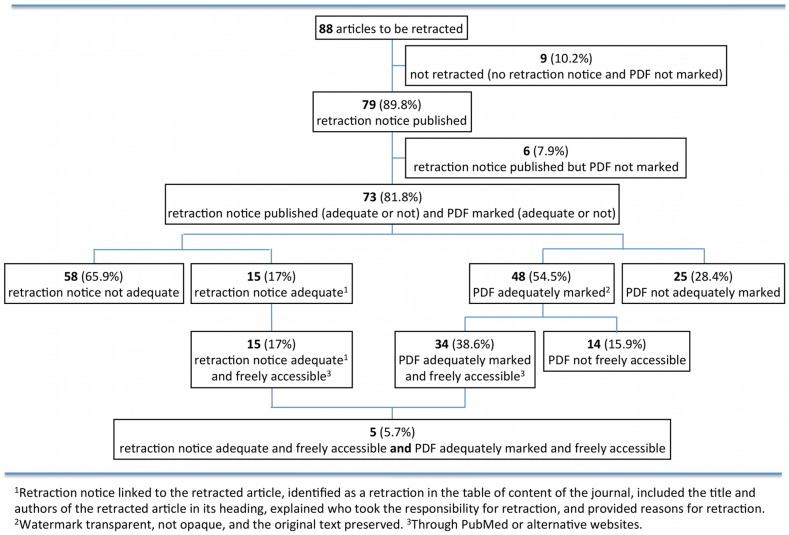
Flow chart. ^1^Retraction notice linked to the retracted article, identified as a retraction in the table of content of the journal, included the title and authors of the retracted article in its heading, explained who took the responsibility for retraction, and provided reasons for retraction. ^2^Watermark transparent, not opaque, and the original text preserved. ^3^Through PubMed or alternative websites.

Five retractions (6%; published in one journal) fulfilled all predefined criteria of adequate retraction (Fig. 1).

#### Retraction notices

No retraction notices had been published for nine of the articles (10%; five journals). Of these five journals, three (publishing four articles) had not published any retraction notice at all, one had published retraction notices for only two of six articles, and one had published a retraction notice for only one of two articles (Table 1). Retraction notices for the remaining 79 articles were published between May 2011 and October 2011.

Each of the 79 retraction notices was linked, in Pubmed, to the retracted article; 55 (63%) notices were clearly identified as “Retractions”, one was labelled “Statement”, one “Erratum”, and 22 (originating from one journal) were not labelled at all and referred to an Editor's Note named: “Notice of retraction”. 76 retraction notices (86%) were listed in the table of contents of the respective journals; in 52 (59%; nine journals) the complete reference of the retracted article was listed in the table of contents, and in 24 (27%; four journals) the retraction was listed in the table of contents but the reference was lacking. Three published retraction notices (two journals) were not listed in the table of contents.

The formats of the retraction notices were consistent within each journal, but differed between journals (Table 1): 53 notices (60%) included the title of the retracted article, and 32 (36%) included the names of the authors in their heading. Twenty notices (23%; four journals) included both the title and the authors in their heading, and 14 (16%) included neither of them in the heading, but included the reference in the text of the retraction notice.

Seventy-one retraction notices (81%) described who had taken responsibility for the retraction. Responsibilities varied across journals. In seven journals (48 retraction notices), the editor-in-chief signed the retractions, in three (ten notices), it was the editor-in-chief with the publisher, and in two (13 notices), responsibility was taken by the editorial board. In three journals (eight notices), there was no indication of who had retracted the article; for example, one stated, *“the following article has been retracted...”*. Reasons for the retraction were explicitly provided in the notices in 13 journals (49 retraction notices); in two journals (30 notices), the notice referred to an editorial that explained the context of the retractions.

Sixty-seven retraction notices (76%) were accessible through Pubmed, nine retraction notices (10%; one journal) were accessible through an alternative weblink (ScienceDirect) but not through Pubmed, and three (3%; two journals) were not freely accessible. Overall, only 15 retraction notices (17%; three journals) were found to fulfil all predefined criteria for an adequate retraction notice (Fig. 1).

#### Retracted articles

**Figure 2:**
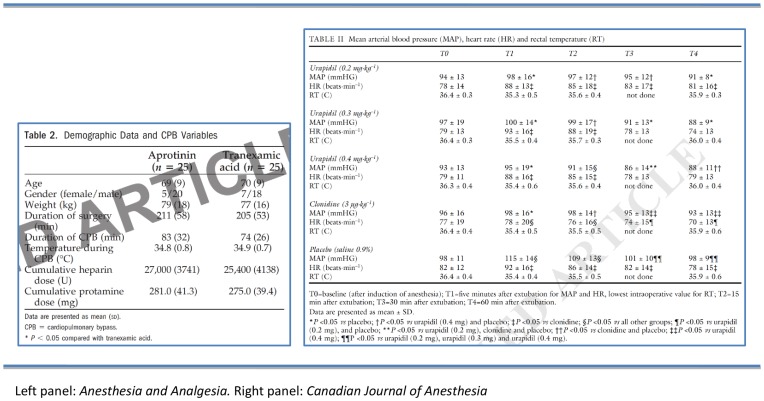
Transparent watermarks. Left panel: *Anesthesia and Analgesia*. Right panel: *Canadian Journal of Anesthesia*.

**Figure 3:**
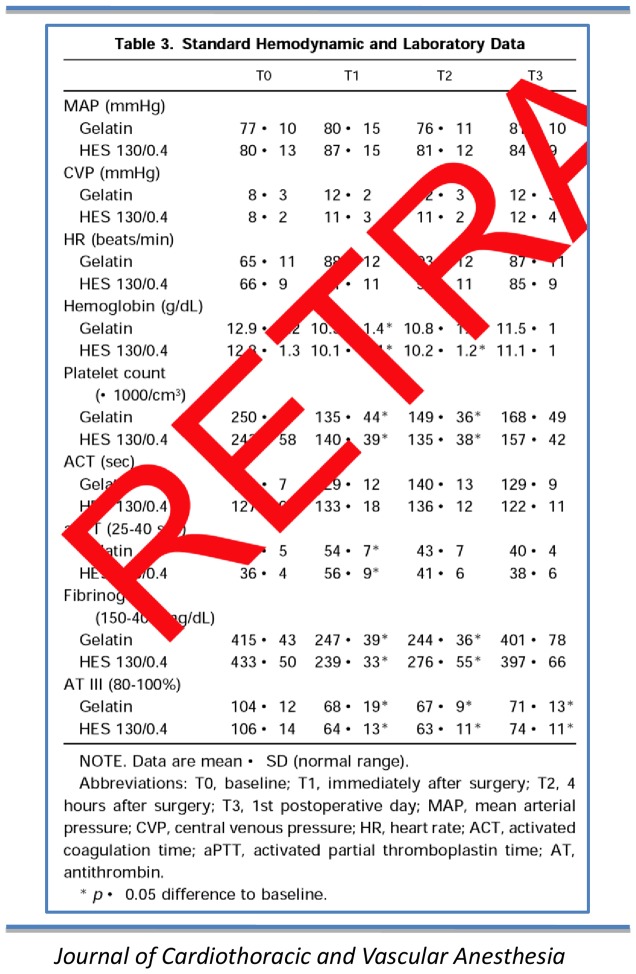
Opaque watermark. *Journal of Cardiothoracic and Vascular Anesth*.

Fifteen articles (17%) were not marked as retracted. Nine of these also lacked a retraction notice. Of the 73 articles (83%) that were marked as retracted, 48 (55%; eight journals) had transparent watermarks although in ten of those (11%; two journals), the watermark was almost invisible (Fig. 2). The other 14 articles (16%; four journals) had opaque marks across all pages that completely obscured parts of text, tables or figures (Fig. 3). Eleven articles (13%; all from one journal) had their entire content (i.e. text, tables, figures, references) deleted.

In one journal, only seven of eight full texts were labelled with a watermark although retraction notices were published for all eight. The article that lacked a watermark had been published by the journal's previous publisher.

Forty-four articles (50%; six journals) were freely accessible through Pubmed, but 11 of those (13%; one journal) had their content completely deleted. Fifteen full text articles (17%) were freely accessible, although through alternative weblinks only; one of them was not marked. Overall, only 34 articles (39%) were both adequately marked and freely accessible (Fig. 1).

#### Explanations from editors-in-chief and publishers

We contacted the editors-in-chief of the journals that had not correctly implemented some or all retractions and asked them for explanations. Two did not respond. One referred to personal health problems that prevented him from accomplishing the task. The others referred us to their publishers. One of these editors cited internal communication problems that led to the omission of one of two retraction notices. One editor explained that it had been the publisher's decision to delete the content of the retracted articles. However, he also challenged the principle that data in retracted articles should be preserved, as he considered these data were false and therefore valueless.

We were able to communicate with three publishers. One was critical of the fact that there was no mechanism within the industry that he knew of for previous publishers to be notified of retractions required by journals that were now produced by a different publisher. Two publishers mentioned legal threats from Boldt's co-authors that prevented them from retracting a total of six articles in three journals.

## Discussion

We followed up the fate of 88 articles that should have been retracted due to the absence of ethical approval. Almost two years after the need for retraction was recognised, following an official investigation, our study shows that the performance of many journals is disappointing. Applying our stringent rules, only five articles (6%; one journal) were adequately retracted. A retraction notice had been published for only 79 (90%) of the articles, and although most retraction notices were listed in the journal's table of contents, 27% of the listings were incomplete. Also, only 48 (54%) full text articles were clearly and correctly marked as retracted; the others were not marked at all, or the text was either completely deleted or covered by an opaque watermark obscuring parts of the original information. A major issue was the lack of free access to both retraction notices and full texts of retracted articles; only 76 (86%) of all retraction notices and 59 (67%) of all retracted articles were freely accessible, and not all of those were freely accessible through PubMed but had to be accessed through alternative websites.

Formal retraction from the literature is the most severe sanction for a published research article [2]. Since science must be self-correcting, retraction of unreliable articles is an essential step for rectifying the scientific knowledge-base [11], although it has been shown that it is probably insufficient to inform the scientific community [12]. Retractions appear to be “unpopular” with both editors and institutions since they may shed doubt on the integrity of science, and on the expertise of the editorial team. However, they demonstrate the determination to maintain the integrity of knowledge and to prevent readers from being misled by unreliable information [10], [13]. What is certain is that retractions are, and will remain, necessary since the safeguards of science including the process of peer review, remain vulnerable to fraud and error. So why did we find such great disparity in the way in which these 88 articles were handled? There may be several explanations.

First, nine (10%) articles have not been retracted at all. There is little agreement on what exactly requires a retraction [8], and lack of ethical approval does not fit into any of the conventional definitions of misconduct such as plagiarism, fabrication, or falsification of data. Therefore, some editors and publishers may be inclined to consider ethical concerns as a minor problem only. NLM advises that articles may be withdrawn because of “pervasive error” or “unsubstantiated or irreproducible data” due to either misconduct or honest error. COPE advises journal editors to retract an article if they have clear evidence that the findings are “unreliable”, and that they should consider retracting a publication if it reports “unethical research”. The 88 articles by Dr. Boldt and co-workers do not clearly fit into any of these categories and there is not yet an agreement on how to handle a clinical research article that may not necessarily be “unethical” *per se*, but that was not officially approved by a competent ethics committee. It is therefore possible that retraction was delayed or prevented because the articles did not fit into one of the “usual” categories requiring retraction. However, this reason was not mentioned by any of the editors who were contacted requesting an explanation and this does not explain why the retractions failed to adhere to published guidelines.

Second, no reliable mechanisms exist to ensure that research articles warranting retraction (e.g. following an appropriate investigation) are actually retracted [7], [10], [14] although the COPE guidelines on cooperation between journals and institutions give some guidance on this [4]. For example, the primary responsibility for investigating possible scientific misconduct rests with the authors' institution. Once an institution has determined that misconduct involving a research publication has occurred, journals are obliged to consider retraction of the work [11]. In the present example, suspicion of scientific misconduct was first raised by journal editors, who then turned to the authors' institution and its ethics committee, asking for an internal investigation. When misconduct was recognised by the competent ethics committee, the editors-in-chief of all journals involved decided to start a joint retraction process. However, the individual retraction processes were handled independently by each journal, and did not follow a clearly defined common procedure.

Third, it remains unclear who should be responsible for retracting an article. In cases of unintentional “honest” error, the responsibility for requiring a retraction rests with the authors. In cases of scientific misconduct, obviously, someone else has to assume this responsibility. It has been claimed that, once an institution has identified fraud or significant error, it is then up to the journals to respond promptly and properly [4], [14]. However, there is no clear guidance about who, within the “journal”, should take this responsibility. Surveys of retractions have shown that this varies and may be the editor-in-chief, the entire editorial board, the publisher, or the owner of the journal, which may be an academic society [6]. Interestingly, retraction notices sometimes specified that an editor-in-chief alone had ordered the retraction, and sometimes there seemed to be an agreement between a publisher and an editor-in-chief or the entire editorial board. Most editors of journals that had failed to correctly retract some or all articles, referred us to their publishers, suggesting that they held them responsible. As long ago as 1990 it was recognised that authors, editors, reviewers, and librarians all needed to be involved in a multifaceted approach to address the continued use of invalid data [15]; interestingly, publishers were ignored at that time. By 2012, adequately dealing with scientific misconduct has become, according to different guidelines, a joint mission for “authors, editors, and publishers” [16]–[18]. The exact responsibility of each actor, however, remains ill defined.

Fourth, recommendations on how full texts of retracted articles should be labelled are also lacking. COPE, for instance, states that retracted articles should not be removed from printed copies of the journal or from electronic archives but their retracted status should be indicated as clearly as possible. The question remains, whether the original content of these articles should be preserved. Some may agree with one of the interviewed editors who argued that the data were false and therefore valueless, so why leave it viewable? An alternative argument may be that scientific data, even when fraudulent or unethical, belongs to the public and may serve future research, for instance, research into fraud, and that the preservation of the historical record, including all faults, mistakes, and corrections, is essential [10]. Indeed, in 1984, when the NLM implemented a policy for identifying and indexing published retractions, they chose to link the notice of retraction to the original article rather than delete the citation to the retracted article, because they felt that removal might affect historical perspective [10]. In the present study, data from 25 retracted articles (28% of retracted articles) had been partially or completely removed; either the contents of the articles were completely deleted, or parts of the underlying text, tables or figures were hidden by opaque watermarks.

Fifth, a further unresolved issue refers to the ultimate control after a retraction has been initiated. Who ensures that an article is adequately retracted, that a notice is published in the journal and indexed in databases such as Pubmed, that the full text article is clearly marked and freely accessible electronically, and that the original information remains visible? When studying various recommendations on how to retract articles [2], it is striking how much responsibility is given to the editors. Several guidelines indicate that editors are responsible for the final decision about retracting material, with or without cooperation of the authors, and additionally that they should ensure that retractions are labelled in such a way that they are identified by bibliographic databases. There seems to be some contradiction here since we found that some editors considered they were powerless against a publisher's decision. Also, current guidelines do not specify who should verify whether the retraction process has been implemented correctly.

Finally, journal editors or publishers may be reluctant to issue retractions and to mark an article because they may fear legal actions by discredited authors. In our example, it was sometimes a publisher that decided not to retract an article because Boldt's co-authors threatened legal action. This highlights, again, the central role of publishers in deciding whether an article is to be retracted or not. Today, the threat of legal action weighs heavily, especially on smaller journals [9], although, according to COPE, authors usually would not have grounds for taking legal action against a journal over the act of retraction if it follows a suitable investigation and proper procedures [2]. COPE also states that journal editors should consider at least issuing an expression of concern if an investigation is underway but a judgment will not be available for a considerable time [2]. None of the journals that failed to retract an article has published such an expression of concern.

Our analysis has some limitations. The main weakness of our study is that it remains descriptive and relates to a single series of papers involving one author and a single investigation into a specific case of lack of evidence of ethical approval, which may not be typical of most retractions; we have not attempted to identify potential “risk factors” for problems in the retraction process. The reason for this is that we had no strong *a priori* hypothesis to test, and the relatively small sample size would have prevented us from performing multivariate analyses. However, our analyses may serve as a basis for future larger studies focusing on retractions due to ethical issues and for other reasons. Indeed, this descriptive cross-sectional study is the first of its kind to describe systematically the disparity of the retraction processes in a uniform context; one common author for each article and a single type of misconduct (lack of ethical approval) was involved. Selection bias is unlikely since we included all articles by Dr. Boldt that had been identified by an official investigation [1]. It cannot be excluded that a few additional studies will eventually require retraction because of lack of ethical approval but it is unlikely that this will change the overall picture. The risk of reporting bias was minimized by having two researchers extract the relevant data separately.

The Boldt case was a shock to the academic world [19]. The impact of this debacle was recognized even outside peri-operative medicine [20]. Perhaps the only positive and encouraging fact was that the editors-in-chief of 18 journals agreed, in a well-orchestrated, committed and overt way, to sign a strong public statement and to retract 88 articles. This organised approach against fraud, across so many journals, was probably unique in the scientific world until then, and it has been cited as a laudable example of how journal editors should play a more active role in the retraction of fraudulent papers [8]. Since, an even larger case of scientific misconduct, necessitating the retraction of 183 articles, has been uncovered [21]. Our study shows that purging the literature of fraudulent or unethical articles remains a technically challenging process and highlights several weaknesses which must be addressed. This is probably more a confirmation than a revelation. We did know that retractions were imperfect, but our study helps to quantify this. Perhaps most fundamentally, there must be clear and universally accepted definitions about what type of articles deserve retraction. We feel strongly that science that has not been approved by a competent ethics committee should fall into this category and we suggest that COPE's wording about “unethical research” should be amended to include this. The roles and responsibilities of the different players must be unambiguously defined, and publishers must not be left out – in fact, they should probably have a central role. The mechanism for retracting articles published by a previous publisher (i.e. after a journal has switched publishers) needs to be resolved. It seems obvious that once a competent independent investigation has provided convincing evidence that an article should be retracted, it is the journal's responsibility to issue a retraction notice, but it remains unclear who must take on that responsibility, the editor or the publisher. We suggest that it should be the publisher's responsibility to adequately mark the full text of the retracted article, using a transparent rather than opaque watermark on each page. Deleting the content of the fraudulent article should be outlawed. Both retraction notices and retracted articles belong to the public domain and should not be hidden behind access barriers or pay walls. After a reasonable time period, it should be verified whether the retraction process has been successfully implemented, although, again, it remains unclear who shall take on that responsibility, but perhaps this role could be taken by the institution that carried out the investigation. And finally, legal safeguards are needed that allow journal editors to retract fraudulent or unethical papers even against the wishes of authors and/or publishers.
